# Cysts of Mesentery

**Published:** 1915-10

**Authors:** 


**Cysts of Mesentery,** Edward G. Jones, Atlanta, Surg., Gyn. & Obs., July, briefly reviews the literature and reports 3 cases: Male aged 4, convulsions and abdominal trouble at intervals since 6th month, operated for partial obstruction. Bilocular chylous cyst 16x8 c.m., 4 feet from ileo-caecal valve, resected with intestine to which it was adherent and upon which it pressed. Death from shock 30 hours later. 2. Male, aged 54, left oblique inguinal hernia 15 years, right 6 years, former irreducible for 2 years. Operation for radical cure revealed serous mesenteric cyst, not exactly located but along small intestine, 1 inch from intestinal attachment, 3 1/2 inches in greatest diameter. Enucleated. Recovery. 3. Male, aged 32, recurrent attacks of pain in right upper quadrant of abdomen, loss of weight, failure of digestion. 3 serous cysts in mesentery of colon near hepatic flexure. Enucleated. Recovery.

